# Trichlorid of Iodin in Dentistry

**Published:** 1919

**Authors:** 


***Trichlorid of Iodin in Dentistry.*** By MILTON J. WAAS, D. D. S. *Dental Cosmos*, October 1918.

Trichlorid of iodin $ICl_3$ is an atomic compound of chlorin and iodin having three atoms of chlorin to one of iodin; it is a heavy reddish brown fuming powder, acid in reaction, freely soluble in water. It is put up and kept in glass vials, and all solutions must be prepared in glass containers; no metal should be used. Distilled water must be used for solutions; the stronger solutions from 1 per cent. up keep for a long time. The three solutions most useful in dentistry are 25 per cent., 1 per cent., and 1 : 1000. The last named should not be kept long.

The 25 per cent. solution is used to dissolve broken-off steel instruments of any sort which may be found in root-canals and cannot be dislodged by mechanical means. Tantalium or platinum pointed instruments or syringes should be used in applying the solution. Several applications may be necessary, if only a small portion of the metal is exposed. This solution is caustic to soft tissues and must not be allowed to pass beyond the apex.

A one per cent. solution may be used in contact with soft tissues, and is very useful for washing out root-canals, fistulous tracts, sockets after extraction, cavities left after apicectomy operations, and pyorrheal pockets. It should be used in an all-glass syringe with platinum point. It is also useful for surface sterilization.

The 1 : 1000 solution, with the addition of saccharin to improve the taste, is " by far the most efficient germicidal mouth wash ever used ". Its use is especially indicated in pyorrheal treatment before the curettement, and for one or two weeks afterwards. Its use should not be continued indefinitely, as it is somewhat acid in reaction.

$ICl_3$ is soluble is chlorcosane, Dakin's new single (paraffin) solvent for dichloramin-T, and such a solution might be useful to seal in an infected root-canal as a dressing.

